# Characterization of optic nerve regeneration using transgenic zebrafish

**DOI:** 10.3389/fncel.2015.00118

**Published:** 2015-04-09

**Authors:** Heike Diekmann, Pascal Kalbhen, Dietmar Fischer

**Affiliations:** Division of Experimental Neurology, Department of Neurology, Heinrich-Heine-University of DüsseldorfDüsseldorf, Germany

**Keywords:** transgenic zebrafish, axon regeneration, GFP, optic nerve regeneration, tissue clearing

## Abstract

In contrast to the adult mammalian central nervous system (CNS), fish are able to functionally regenerate severed axons upon injury. Although the zebrafish is a well-established model vertebrate for genetic and developmental studies, its use for anatomical studies of axon regeneration has been hampered by the paucity of appropriate tools to visualize re-growing axons in the adult CNS. On this account, we used transgenic zebrafish that express enhanced green fluorescent protein (GFP) under the control of a GAP-43 promoter. In adult, naïve retinae, GFP was restricted to young retinal ganglion cells (RGCs) and their axons. Within the optic nerve, these fluorescent axons congregated in a distinct strand at the nerve periphery, indicating age-related order. Upon optic nerve crush, GFP expression was markedly induced in RGC somata and intra-retinal axons at 4 to at least 14 days post injury. Moreover, individual axons were visualized in their natural environment of the optic nerve using wholemount tissue clearing and confocal microscopy. With this novel approach, regenerating axons were clearly detectable beyond the injury site as early as 2 days after injury and grew past the optic chiasm by 4 days. Regenerating axons in the entire optic nerve were labeled from 6 to at least 14 days after injury, thereby allowing detailed visualization of the complete regeneration process. Therefore, this new approach could now be used in combination with expression knockdown or pharmacological manipulations to analyze the relevance of specific proteins and signaling cascades for axonal regeneration *in vivo*. In addition, the RGC-specific GFP expression facilitated accurate evaluation of neurite growth in dissociated retinal cultures. This fast *in vitro* assay now enables the screening of compound and expression libraries. Overall, the presented methodologies provide exciting possibilities to investigate the molecular mechanisms underlying successful CNS regeneration in zebrafish.

## Introduction

Adult teleosts have the remarkable ability to functionally regenerate severed axons in the central nervous system (CNS), for example after lesion of the optic nerve or spinal cord (Stuermer et al., 1992; Bernhardt et al., 1996; Becker and Becker, 2007). This stands in stark contrast to the very limited restorative capability of mammals, including humans, upon CNS injuries (Schwab et al., 1993; Fawcett, 2006; Berry et al., 2008; Fischer and Leibinger, 2012). Fish therefore offer the exciting possibility to study molecular mechanisms underlying successful axon regeneration that might potentially be developed into new treatment options for mammals (Zon and Peterson, 2005; Goessling and North, 2014; Patten et al., 2014; Rennekamp and Peterson, 2015). Early classic studies have been predominantly performed in goldfish, but lately the zebrafish is gaining more attention in CNS regeneration research (Stuermer et al., 1992; Cameron, 2000; Veldman et al., 2007; Graciarena et al., 2014; Lewis and Kucenas, 2014; Liu et al., 2014). Zebrafish are an established vertebrate model for developmental studies and have the advantages of experimental and genetic accessibility, transgenic and mutant availability and ease of gene expression manipulation. Their high intrinsic capacity for long-range axonal regrowth is exemplified in the restoration of the visual projection. Upon crush or complete transection of the optic nerve, retinal ganglion cells (RGCs) survive and their severed axons regrow through the optic nerve and tract to topographically re-innervate their respective targets in the brain, leading to functional recovery 2–4 weeks after injury (Stuermer et al., 1992; Bernhardt, 1999; McDowell et al., 2004).

The regenerative state of axotomized zebrafish RGCs is indicated by the expression of a number of regeneration-associated genes (Bernhardt et al., 1996). Among these, growth associated protein 43 (GAP-43) has long been recognized as a hallmark of axonal growth (Skene, 1989; Benowitz and Routtenberg, 1997; Bormann et al., 1998; Kaneda et al., 2008). Careful analysis of its promoter revealed the requirement of different elements for the induction of GAP-43 expression during developmental axonal growth and regeneration, respectively (Udvadia et al., 2001; Kusik et al., 2010). Recently, transgenic zebrafish have been generated that express a cell membrane-tagged version of the fluorescent reporter green fluorescent protein (GFP) under the control of the compact *Takifugu rubripes* GAP-43 promoter (Tg(fgap43:GFP); Udvadia, 2008). These fish induce GFP temporarily in various growing axons during development similar to native GAP-43 expression and in RGC axons upon injury (Udvadia, 2008). As this transgenic fish could be particularly suitable to visualize regenerating axons, we sought to further characterize the injury-induced GFP expression and to establish it as a model for future axon regeneration studies. In this manuscript, we analyzed the expression of GFP in adult naïve zebrafish RGCs as well as its induction upon optic nerve crush. In addition, introduction of tissue clearing and subsequent confocal microscopy enabled us to accurately visualize and characterize the time course of axonal regeneration in the whole optic nerve. Finally, we established a reliable RGC culture approach for pharmacological *in vitro* axon regeneration studies using this transgenic fish. Altogether, this combination of a regeneration-induced transgenic axon label with visualization and culturing methods will facilitate the future use of adult zebrafish in RGC axon regeneration research.

## Materials and Methods

### Zebrafish

Adult, 4-8 months old, homozygous Tg(GAP43:GFP) zebrafish, in the text referred to as GAP43:GFP, were used for all experiments (Udvadia, 2008). Zebrafish were reared and kept in the zebrafish facility of the University of Düsseldorf on a 14 h light/10 h dark cycle under standard conditions (Westerfield, 1989). All experimental procedures were approved by the local animal welfare committee in Recklinghausen and conducted in compliance with federal and state guidelines for animal experiments in Germany. No experimental differences were observed between male and female zebrafish.

### Dissociated Retinal Cell Cultures

Tissue culture plates (4-well-plates; Nunc) were coated with poly-D-Lysine (0.1 mg/ml, molecular weight 300,000 Da) (Sigma), rinsed with distilled water and air-dried. Zebrafish were sacrificed by immersion in MS222 (0.4 mg/l) and decapitation. Retinae were rapidly dissected from the eyecups and incubated in a digestion solution containing papain (10 U/ml, Worthington) and L-cysteine (0.3 µg/ml, Sigma) in L15/salt solution (12.5% salt solution: 10 mM D-glucose, 1.26 mM CaCl_2_, 32 mM Hepes, pH 7.5/87.5% L15; Invitrogen) at room temperature for 40 min. They were then rinsed with L15/salt solution and triturated in 2 ml fish medium (2% FBS (Invitrogen), 0.2 mg/ml penicillin/streptomycin (Biochrom) in L15/salt solution). Dissociated cells were passed through a cell strainer (40 µm; Falcon) and counted using a TC10 Automated Cell Counter (BioRad). Approximately 2.5 × 10^4^ cells were added to each well. In some wells, 2 ng/ml mouse CNTF (Peprotech) was added to the culture medium. Cultures were incubated at 27.5°C in a humidified incubator. Neurite growth was determined after 4 days in culture by fixing the cells in 4% paraformaldehyde (PFA) in PBS for 30 min at room temperature. RGCs with regenerated neurites were photographed under a fluorescent microscope (200X, Observer.D1, Zeiss) and neurite length determined using ImageJ software. Mean neurite length was calculated by dividing the sum of neurite length by the number of RGCs with regenerated neurites per well. Data are given as the mean ± SEM of six replicate wells from two independent experiments. The significance of intergroup difference was evaluated using Student’s *t*-test.

### Optic Nerve Crush

For surgery, zebrafish were anesthetized by immersion in MS222 (0.18 mg/l; Sigma). The eye was slightly pulled out of its orbit to expose the optic nerve. Taking care to spare the ophthalmic artery, the optic nerve was intra-orbitally crushed ~0.5 mm behind the eye for 5 s using jeweler’s forceps (FST), as described previously (Bormann et al., 1999; Liu and Londraville, 2003).

### Retinal Flatmounts

At various times after optic nerve crush, zebrafish were sacrificed by prolonged immersion in MS222 (0.4 mg/l) and decapitation. Eye(s) were removed and retinae dissected as described previously (Zou et al., 2013; Raymond et al., 2014). Incisions were made at four points around the circumference and the retina mounted on a blackened nitrocellulose filter (0.45 µm; Sartorius Stedin Biotech). Retinae were then fixed in 4% PFA/PBS for 30 min at room temperature and photographed on an inverted microscope (10× objective, Observer.D1, Zeiss) equipped with an AxioCam MR3 camera (16 bit grayscale) and various exposure times as indicated. At least three retinae were analyzed per time point with comparable results.

### Retinal Cross Sections

Zebrafish were sacrificed by prolonged immersion in MS222 (0.4 mg/l) and decapitation. Eye(s) were removed and fixed in 4% PFA/PBS at 4°C overnight. Subsequently, eyes were immersed in 30% sucrose and embedded in Tissue-Tek (Sakura). Frozen sections (14 µm) were cut on a CM3050S cryostat (Leica), thaw-mounted onto glass slides (Superfrost plus, ThermoFisher) and stored at −80°C until further use.

### Immunohistochemistry

Retinal flatmounts and retina cross sections were permeabilized with 100% Methanol for 5 min at room temperature. After blocking with 2% BSA/5% donkey serum/PBS, they were incubated with either choline acetyl transferase (CHAT; 1:100; Millipore) or acetylated tubulin (1:1000; Sigma) antibodies overnight at 4°C. After several washes with PBS, primary antibodies were visualized with anti-mouse or anti-goat secondary antibodies conjugated to Alexa Fluor 488 or Alexa Fluor 594 (1:1000; Molecular Probes).

### Optic Nerve Clearing

At various times after optic nerve crush, zebrafish were sacrificed by prolonged immersion in MS222 (0.4 mg/l) and decapitation. The lower jaw and the gills were removed and the eyes pulled slightly out of their sockets to stretch the optic nerves. The head was then fixed in 75 mM Lysine/2% PFA/10 mM NaIO_4_ overnight at 4°C. After fixation, the optic nerves were dissected with the retina attached and placed into FocusClear solution (BioRad) overnight for clearing. They were embedded in MountClear and scanned using a confocal microscope (LSM510, Zeiss). At least three optic nerves were analyzed per time point with comparable results.

## Results

### Anatomy of the Zebrafish Visual System

**Figure 1:**
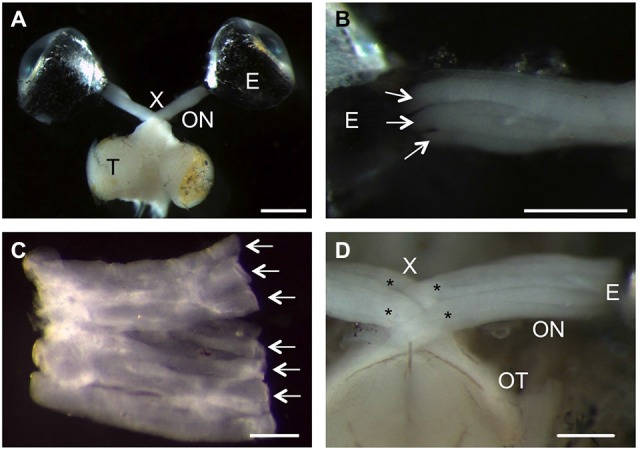
**Anatomical peculiarities of the zebrafish visual system. (A)** Dorsal view of an isolated zebrafish visual system. Retinal ganglion cell (RGC) axons project from the right eye (E) into the optic nerve (ON, fixed in a stretched position), through the optic chiasm (X) and the optic tract into the contralateral optic tectum (T). Scale bar = 1 mm. **(B)** The ribbon-like structure of the zebrafish optic nerve is apparent as RGC axons exit the eye (E) within discrete strands (arrows). Scale bar = 500 µm. **(C)** Upon dissection, the zebrafish optic nerve can be flattened into a sheet of adjacent axon strands (arrows). Scale bar = 200 µm. **(D)** Ventral view of the optic chiasm. The optic nerves (ON) each split into two larger bundles (stars) that intercalate at the chiasm (X). Scale bar = 250 µm.

The zebrafish has recently gained increased attention in CNS regeneration research, with the tacit assumption that the anatomy of its visual system is identical to the previously used goldfish. However, a few peculiarities became apparent upon dissection as illustrated in Figure 1. The overall projection pattern was comparable to the goldfish, with RGCs of one eye sending their axons through the optic nerve and tract to the contralateral optic tectum (Figure 1A). Closer inspection revealed a ribbon-like structure of the zebrafish optic nerve, with RGC axons segregating into discrete strands (Figures 1B,C). Tearing of the dural sheet around the nerve upon dissection allowed flattening of the nerve into a continuous sheet, with axon strands lying adjacent to each other (Figure 1C). Although RGC axons from both eyes crossed completely at the optic chiasm, each optic nerve split into two bundles that interdigitate with the respective bundles of the other side (Figure 1D; Mogi et al., 2009). Therefore, zebrafish optic nerves form a more complicated decussation pattern than goldfish, which might influence the growth pattern of regenerating axons.

### Retinal GFP Expression in Naïve GAP43:GFP Zebrafish

**Figure 2:**
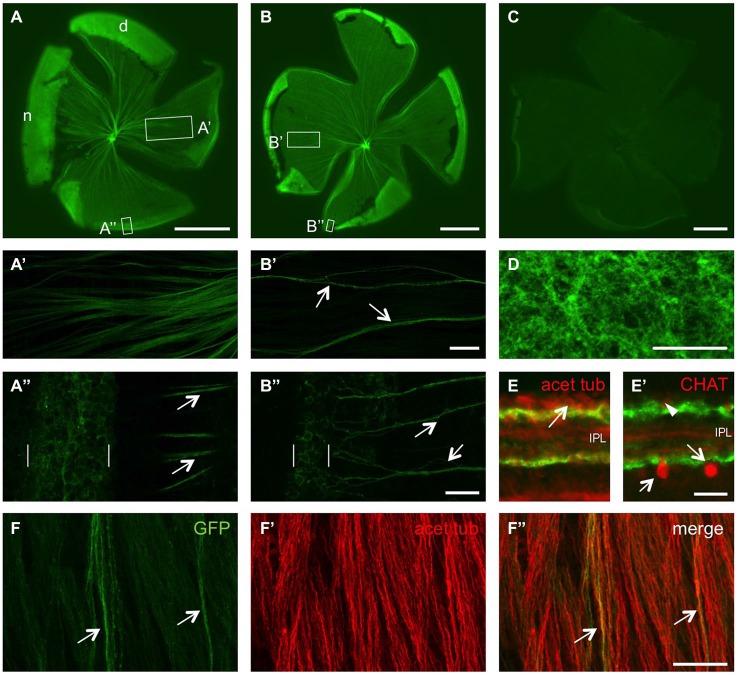
**Size- and age-dependent retinal GFP expression in naïve GAP43:GFP zebrafish. (A)** GFP expression is detected in quite a few RGC axons of a retinal flatmount from a 1.6 cm long, 4 month old zebrafish. **(B)** Only a subset of retinal axons showed GFP expression in a retinal flatmount of a 3 cm long, 4 month old zebrafish. **(C)** Hardly any GFP expression is detected in a retinal flatmount of a 4 cm long, 8 month old zebrafish. Retinae are orientated with dorsal (d) up and nasal (n) to the left. Exposure time = 150 ms. Scale bar = 500 µm. **(A’,A”,B’,B”)** Higher magnifications of the boxed areas in **(A,B)**, respectively, using maximum intensity projections of confocal stacks. GFP is expressed in more RGC axons in smaller/younger retinae (compare **A’** to the few axon fascicles (arrows) in **B’**), originating from a broader proliferative marginal zone (brackets) in the retinal periphery (compare **A”** with **B”**). Retinal periphery is to the left. Scale bar = 50 µm **(A’,B’)** and 20 µM **(A”,B”)**, respectively. **(D)** Dendritic arbors of RGCs are GFP-positive throughout the retina in a flatmount of a naïve GAP43:GFP zebrafish. Maximum intensity projection of 4 Z-sections from underneath the RGC layer. Scale bar = 20 µm. **(E)** Retinal cross sections of naïve GAP43:GFP zebrafish reveal dendritic GFP expression (green) in two separate bands within the inner plexiform layer (IPL). Immunohistochemical co-staining with acetylated tubulin (acet tub, red) identifies RGC somata (arrow) adjacent to the upper band (see also Figure 5G). **(E’)** GFP expression is neither detected in cholinergic amacrine cells located in the inner plexiform layer (arrows) or displaced in the ganglion cell layer (arrowhead) nor in their dendritic arbors as identified by choline acetyltransferase (CHAT) staining (red; see also Figure 5H). **(F)** Co-immunostaining of a retinal flatmount from naïve GAP43:GFP zebrafish with acetylated tubulin antibody **(F’)** reveals all RGC axons while GFP (green) is only expressed in a small subset of retinal axons (arrows). **(F”)** shows the merged picture. Scale bar = 25 µm.

In order to characterize GFP induction after optic nerve injury in adult GAP43:GFP zebrafish, we first looked at the status quo expression in naïve retinae. In retinal flatmounts of ~4 month old fish, a subset of RGC axons was clearly labeled (Figures 2A,A’,B,B’). Depending on the size of the zebrafish/eye, GFP expression was detected in more (small fish, Figure 2A) or less (larger fish, Figure 2B) axons, which was particularly striking on confocal images of the respective retinae (Figures 2A’,B’). While retinae from animals younger than 4 months contained GFP-positive axons comparable to Figure 2A (data not shown), only very few labeled axons were observed in 8 month old retinae (Figure 2C). At the retinal periphery, GFP was expressed in RGC somata, with more GFP-positive neurons in smaller/younger zebrafish than bigger/older fish (compare Figures 2A”,B”), correlating with the differential number of fluorescent axons (Figures 2A’,B’). Nevertheless, GFP containing axons only comprised a small subset of all axons within the naïve adult retina as visualized by immunohistochemical co-staining with acetylated tubulin antibody (Figure 2F). Although GFP was only detected in RGC somata at the retinal periphery, but not the center, their dendritic arbors in the inner plexiform layer (IPL) were labeled throughout the whole retina (Figures 2D,E). On retinal cross sections, these fluorescent dendrites were visible in two separate bands, adjacent to the RGC layer and the inner nuclear layer, respectively, without intermingling with the dendritic arbors of cholinergic amacrine cells (Figures 2E,E’). Therefore, GFP expression is confined to young, differentiated RGCs in the adult naïve zebrafish retina.

### Retinal Cell Cultures from Transgenic GAP43:GFP Zebrafish

**Figure 3:**
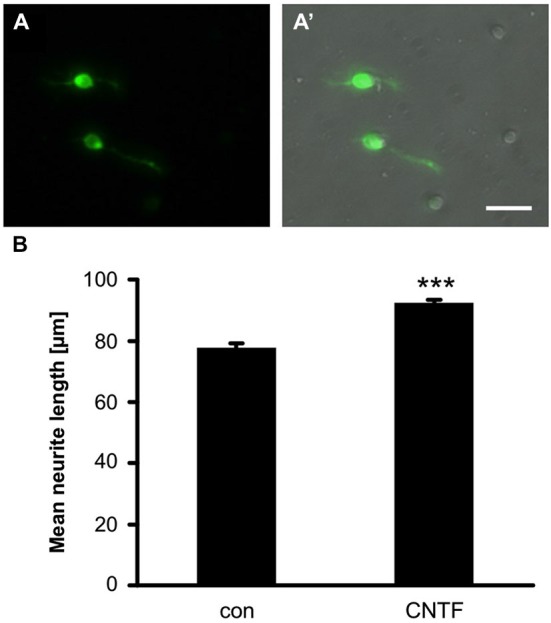
**GFP expression in cultered RGCs. (A)** GFP is expressed in somata and axons of dissociated RGCs at 4 days in culture. **(A’)** Merged view of bright-field and fluorescent image. Scale bar = 100 µm. **(B)** Quantification of neurite length per RGC in retinal cultures of GAP43:GFP zebrafish. Addition of 2 ng/ml CNTF significantly induced neurite growth. Data represent means ± SEM of 6 wells from two independent experiments. Treatment effects (asterisks): *p* < 0.001.

Next, we investigated whether GFP might be expressed in dissociated RGCs, as currently no antibody has been described to unequivocally identify these cells in dissociated cultures. As a first step, we established a protocol to isolate and culture zebrafish RGCs (see Materials and Methods). RGCs were morphologically distinguished by their round to slightly oblong shape, relatively large, phase-bright somata and lengthy neurites after 4 days in culture (Figure 3A’; Ishida and Cohen, 1988). Indeed, a bright fluorescent label was only observed in this cell type (Figure 3A). Therefore, GFP expression driven by the GAP43 promoter is a reliable way to distinguish dissociated RGCs in culture.

GFP was also detected in RGC neurite processes, which allowed the rapid and reliable quantification of neurite outgrowth without the need for immunological staining (Figure 3B). To assess the feasibility of pharmacologically manipulating axon growth *in vitro*, some of the dissociated RGCs were treated with recombinant CNTF, which has previously been shown to increase neurite growth of dissociated rodent RGCs (Müller et al., 2007; Leibinger et al., 2012) and zebrafish retina explants (Elsaeidi et al., 2014). CNTF treatment indeed induced a moderate, but highly reproducible and significant increase in neurite growth (Figure 3B). Therefore, this fast and reliable *in vitro* assay presents a new way to study regeneration-relevant mechanisms and could be used to screen compound and expression libraries to identify molecules underlying successful CNS regeneration in zebrafish.

### GFP Expression in Optic Nerves of Naïve GAP43:GFP Zebrafish

**Figure 4:**
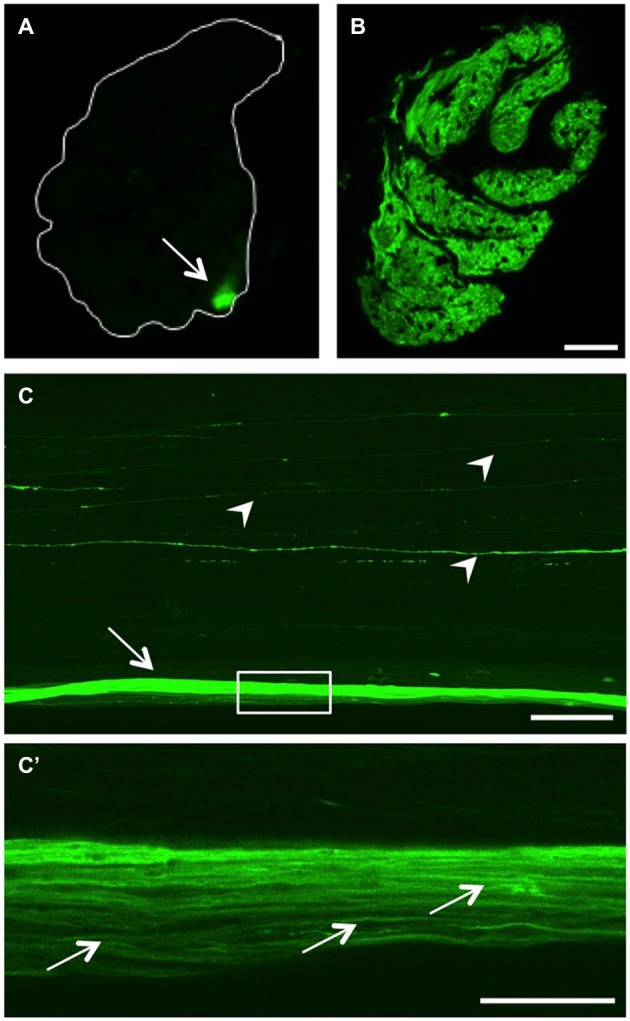
**GFP expression in the optic nerve. (A)** Transverse section of the optic nerve reveals GFP expression in a discrete bundle in the periphery (arrow). The white line indicates the outline of the nerve. **(B)** At 7 days after injury, regenerating RGC axons across the whole optic nerve transverse section are GFP-positive. Scale bar = 25 µm. **(C)** Longitudinal view of a cleared, naïve wholemount optic nerve (maximum intensity projection of a confocal stack). In addition to the labeled peripheral axon bundle (arrow), single GFP-positive axons are visible throughout the optic nerve (arrowheads). Scale bar = 200 µm. **(C’)** Higher magnification of the boxed area in **(C)** using one Z-section of a cleared, naïve wholemount optic nerve reveals single axons within the peripheral bundle (arrows). Scale bar = 50 µm.

As we are particularly interested in studying axonal regeneration *in vivo*, we also sought an appropriate method to visualize single axons within the visual projection. At first, we generated cross sections of optic nerves isolated from either naïve or injured 7 month old GAP43:GFP zebrafish (Figures 4A,B). In the naïve optic nerve, GFP-positive axons originating from the retinal circumference cluster in a compact bundle in the periphery of the nerve. This finding indicates the sorting of retinal axons at the nerve head, which leads to an age-related order within the nerve (Figure 4A). In comparison, all axons across a nerve transverse section were labeled at 7 days post injury, which distinctly visualized the pleated, ribbon-like organization of zebrafish optic nerve (Figure 4B). However, tissue preservation and resolution was not high enough to discern single axons in these preparations.

On this account, we established a method to visualize fluorescent axons in wholemount optic nerve preparations (Figure 4C). Using this approach, the GFP-positive peripheral axon bundle observed on transverse sections (Figure 4A) was visible along the length of the naïve optic nerve (arrow in Figure 4C). In addition, single fluorescent axons coursing through more centrally located nerve strands were detected (arrowheads), that were not readily discernable on cross sections. Upon higher magnification, individual axons were identified within the GFP-labeled peripheral axon bundle (Figure 4C’), indicating superior resolution compared to nerve cross sections (Figure 4A). Evidently, RGC axons do not run absolutely straight within the optic nerve (Figure 4C’). However, their respective paths through the length of the optic nerve could be traced using adjacent confocal Z-sections (data not shown). Therefore, confocal scanning of optic nerve wholemounts should enable adequate and detailed visualization of the plethora of regenerating fish RGCs axons (see below).

### Retinal GFP Expression Upon Optic Nerve Crush

**Figure 5:**
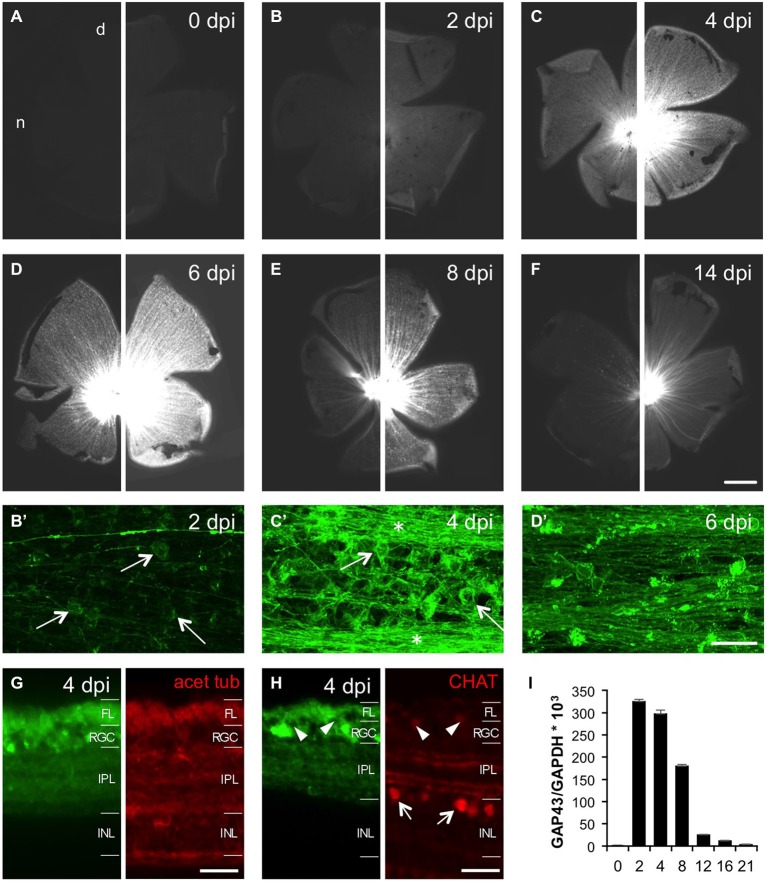
**Time course of optic nerve crush-induced retinal GFP expression. (A–F)** Retinal flatmounts of 8 months old GAP43:GFP zebrafish at 0, 2, 4, 6, 8 and 14 days post injury (dpi), respectively. A retina from an uninjured zebrafish was included to enable direct comparison with experimental retinae. To facilitate the visualization of different GFP expression levels, pictures of the same retina are presented at two different exposure times (30 ms on the left, 100 ms on the right). GFP expression is strongest at 6 dpi. Retinae are orientated with dorsal (d) up and nasal (n) to the left. Scale bar = 500 µm. **(B’–D’)** Higher magnifications of the respective retinal wholemounts using maximum intensity projections of confocal stacks reveal GFP expression in RGCs (arrows), but not yet intraretinal axons (except for the young growing axons) at 2 dpi **(B’)**. At 4 dpi, GFP is strongly expressed in RGCs (arrows) and their axons (stars) throughout the retina **(C’)**. GFP expression in RGCs is already decreasing at 6 dpi **(D’)**. Retinal periphery is to the left. Brightness and contrast were adjusted independently to visualize single axons. Scale bar = 25 µm. **(G)** Cross section of a 4 dpi retina from GAP43:GFP zebrafish co-stained with acetylated tubulin (red) reveals GFP induction (green) in RGCs and their axons in the fiber layer (FL) after optic nerve injury. The dendritic GFP label is dispersed throughout the entire inner plexiform layer (IPL) (compare to Figure 2E). INL = inner nuclear layer. Scale bar = 20 µm. **(H)** Retinal cross section co-stained with choline acetyltransferase (CHAT, red). Retinal GFP induction (green) is restricted to RGCs as cholinergic amacrines in the inner nuclear layer (INL) or displaced in the RGC layer (arrowheads) are not labeled (compare to Figure 2E’). **(I)** Quantitative real time PCR of retinal Gap43 expression relative to GAPDH at various times after optic nerve crush as indicated. Overall, GFP expression closely mirrors GAP-43 induction after optic nerve injury. Values represent the mean of four retinae per group from two independent experiments.

To avoid possible confusion of young, growing axons with injured/regenerating ones, we focused our analysis of GFP induction upon optic nerve injury on 8 month old GAP43:GFP zebrafish, which showed lowest *status quo* expression (see Figure 2). At first, we characterized axotomy-induced GFP expression by preparing retinal flatmounts at various times after optic nerve crush (Figures 5A–F). Two days after injury (2 dpi), GFP was already slightly induced in RGC somata, but not yet in intra-retinal axons compared to naïve retina (Figures 5A,B). At 4 dpi, GFP was strongly expressed in RGC somata and axons throughout the entire retina (Figures 5C,C’). While the highest GFP-labeling of intraretinal axons was observed at 6 dpi (Figure 5D), expression in the cell bodies was already declining at this time point (Figure 5D’). Although GFP expression was further decreasing thereafter, RGC axons were still clearly visible at 8 dpi and 14 dpi (Figures 5E,F). Therefore, injured axons are fluorescently labeled throughout the entire regeneration process in these transgenic zebrafish.

In addition to flatmount preparations, we confirmed RGC-specific GFP induction on retinal cross sections (Figures 5G,H). At 4 dpi, GFP expression was detected in RGCs and their axons in the fiber layer as determined by co-staining with acetylated tubulin (Figure 5G). No GFP was detected in cholinergic amacrine cells, which were identified by choline acetyltransferase co-staining (Figure 5H), or in deeper retinal layers. Interestingly, the dendritic GFP label was no longer confined to two discrete bands, but rather dispersed throughout the entire inner plexiform layer after optic nerve injury (Figures 5G,H). Overall, the time course of retinal GFP expression closely mirrored GAP-43 induction after optic nerve injury as detected by quantitative real time PCR (Figure 5I). Therefore, GFP expression driven by the GAP43 promoter unequivocally identifies regenerating RGCs.

### Time Course of Axonal Regeneration in the Injured Optic Nerve

**Figure 6:**
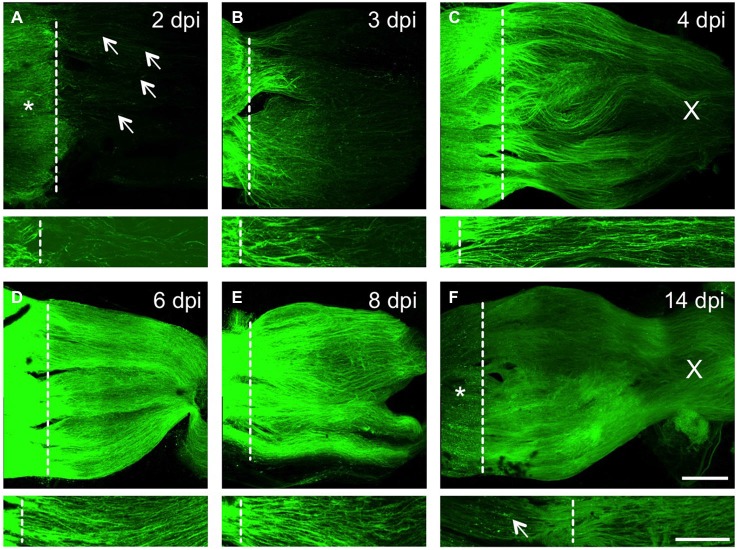
**Time course of axonal regeneration in the injured optic nerve. (A–F)** Maximum intensity projections of confocal scans of cleared, naive wholemount optic nerves of GAP-43:GFP zebrafish at 2, 3, 4, 6, 8 and 14 days post injury (dpi), respectively. The lesion site is indicated with a dashed line, proximal is to the left. Scale bar = 200 µm. Lower pictures depict higher magnifications from one Z-section of the respective optic nerves. In **(A–C)**, these close ups were increased in brightness and contrast to visualize single axons. Scale bar = 100 µm. **(A)** Injured RGC axons strongly express GFP proximal to the lesion site (asterisk) already at 2 dpi. Some axons have started to regrow into the optic nerve (arrows). **(B)** Significantly more axons are regenerating at 3 dpi, with the majority reaching half-distance towards the optic chiasm. **(C)** Even more RGC axons are regenerating in the optic nerve at 4 dpi, with axons already passing through the optic chiasm (X). **(D)** The optic nerve is filled with regenerating RGC axons at 6 dpi, leading to further increased GFP expression. **(E)** Strong axonal GFP expression is still detected at 8 dpi. **(F)** GFP expression is reduced proximal of the lesion site (star) and appears punctuated (arrow) at 14 dpi. The distal part of the regenerating axons, however, is still strongly labeled and individual axons can be identified.

Finally, we studied the time course of axonal regrowth and GFP expression in the injured optic nerve using our previously established wholemount preparations (see Figure 4C). Significant GFP fluorescence was detected proximal to the lesion site as early as 2 dpi and a few pioneering axons had already regenerated up to ~400 µm into the distal optic nerve at this time point (Figure 6A). By 3 dpi, the number as well as the length of regenerating axons increased, with the fluorescent signal now stretching across the entire width of the optic nerve (Figure 6B). The extent of regeneration further progressed by 4 dpi and quite a few axons had already grown past the optic chiasm (Figure 6C). Beyond 6 dpi, the previously injured optic nerve was completely filled with regenerating axons, as indicated by strong GFP fluorescence (Figures 6D,E). Nevertheless, individual axons were still distinguishable on single confocal Z-sections (lower pictures in Figures 6D,E). At 14 dpi, the fluorescence proximal to the lesion site appeared somewhat punctuated and reduced in intensity (Figure 6F), indicating commencing down-regulation of GFP. However, the lesion site and the regenerated distal part of the axons were still clearly discernable (lower picture in Figure 6F). Therefore, re-growing axons can be visualized at high resolution in their natural environment throughout the whole regeneration process. In combination with genetic and pharmacological manipulations, this new approach should now enable more detailed analysis of molecular mechanisms of axonal growth and guidance underlying the successful regeneration of the injured zebrafish CNS.

## Discussion

The current study characterizes GAP43:GFP zebrafish with respect to GFP expression in the adult naïve as well as injured visual system as a means to establish this transgenic line as a tool for axon regeneration studies. Although the remarkable regenerative ability of fish CNS neurons has been known for a long time (Sperry, 1948; Attardi and Sperry, 1963), the zebrafish is only rather recently attracting interest in this area as most classic studies have been performed in goldfish (Murray, 1976; Easter et al., 1981, 1984; Stuermer et al., 1992; Liu and Londraville, 2003). However, anatomical descriptions of its visual system are still rather sparse. Upon dissection, we noticed the ribbon-like structure of zebrafish optic nerves, which has been described for other teleosts (Tapp, 1973; Anders and Hibbard, 1974; Scholes, 1979; Rusoff, 1984; Maggs and Scholes, 1986), but is different from the discrete fascicles in the goldfish optic nerve (Easter et al., 1981; Bunt, 1982). Another obvious anatomical difference between gold- and zebrafish presents itself at the optic chiasm. In goldfish, the optic nerve decussates completely without pre-determined laterality (either the left or the right nerves runs dorsally; (Roth, 1979; Mogi et al., 2009). In contrast, each zebrafish optic nerve splits into two bundles that interdigitate with the respective bundles of the other side at the optic chiasm. We even observed branching of optic nerves into more than four interdigitating bundles in zebrafish obtained from local pet stores (data not shown; see also Mogi et al., 2009). The relevance of this anatomical peculiarity is currently unclear, but it might potentially restrict the occurrence of path-finding errors upon regrowth.

The teleost retina grows throughout much of the fish’s life by continuously differentiating new neurons in concentric annuli at the retinal margin (Johns, 1977; Meyer, 1978). Therefore, the GFP-positive RGCs we observed in the retinal periphery of naïve adult GAP43:GFP zebrafish likely represent recently differentiated RGCs. Since these RGCs are still growing axons from the retina towards the optic tectum (Easter et al., 1981) and GAP-43 expression is closely correlated with axon growth (Skene, 1989; Benowitz and Routtenberg, 1997), it is rather intuitive to find GAP43 promoter-driven GFP in this retinal subpopulation. Nevertheless, this is, to our knowledge, the first description that a protein is expressed in young RGCs, but not in retinal progenitor cells in the peripheral germinal zone as it has been described, for example, for activated leucocyte cell adhesion molecule (ALCAM) and α1tubulin (Laessing et al., 1994; Goldman et al., 2001). Therefore, the easily detectable GFP expression could serve as a new tool to visualize or to isolate young RGCs within the adult naïve living retina, which was previously only possible with more laborious metabolical labeling approaches (Johns, 1977; Johns and Easter, 1977). We detected a wider zone of GFP-expressing RGCs at the peripheral margin of retinae from smaller/younger zebrafish. This finding is consistent with their higher overall growth rate, which depends, among other factors, on fish age as well as nutrient availability and population density (Johns, 1981). Since a membrane-tagged version of GFP is expressed in these transgenic fish, not only RGCs, but also their growing axons are clearly labeled (Udvadia, 2008). Accordingly, more GFP-positive axons were detected in naïve retinae of supposedly faster growing zebrafish.

In an attempt to trace fluorescent axons outside the retina throughout the visual system, we introduced tissue clearing of wholemount optic nerves in combination with confocal microscopy, which has previously been applied to rodent tissue (Fu et al., 2009). Using this approach, we were able to visualize a small bundle of GFP-positive axons at the periphery of the naïve optic nerve. These labeled axons are the ones observed in retinal flatmounts and originate from the retinal circumference. Previous reports describe an age-related order of RGC axons within optic nerves of various fish species (Scholes, 1979; Rusoff and Easter, 1980; Bunt, 1982; Dunn-Meynell and Sharma, 1988). In the sheet of a ribbon-like optic nerve of cichlid fish, oldest axons from the central retina reportedly bundle in a strand along one edge, whereas youngest axons from the retinal periphery cluster at the opposite edge (Scholes, 1979). This anatomy is consistent with our findings in zebrafish, but contrasts the rather topographical order in goldfish (Rusoff and Easter, 1980; Bunt, 1982). Accordingly, each age-related strand is predicted to contain axons from RGCs in a given annulus within the retina. Future studies need to address this hypothesis, for instance by selectively severing single optic nerve strands and detection of axotomy-induced GFP expression within the connected RGCs of the respective retinae of GAP43:GFP zebrafish. It is currently unknown whether the single fluorescent axons we observed coursing through more centrally located ribbons might correspond to specialized RGCs, potentially projecting to minor targets outside of the optic tectum.

We were particularly interested in the transgenic GAP43:GFP zebrafish as a tool for the detailed visualization of regenerating axons. Retinal flatmount preparations confirmed fast and marked GFP induction in RGC somata upon optic nerve injury. In addition, GFP was also detected in intraretinal axons, likely due to its targeting to cell membranes by the GAP43 N-terminus (Udvadia, 2008), which offers an advantage to previously generated transgenic fish with predominantly cell restricted GFP expression (e.g., Goldman et al., 2001; Poggi et al., 2005). The time course of GFP expression closely mirrored the induction of Gap-43 (see also Bormann et al., 1998) and lasted until at least 14 dpi, thereby corresponding to the entire regenerative growth phase (Kaneda et al., 2008). Axotomy-induced GFP fluorescence was restricted to the fiber, RGC and inner plexiform layers and absent from cholinergic amacrine cells, confirming RGC specificity. Interestingly, GFP was also expressed in RGC dendritic arbors throughout the entire naïve retina, possibly indicating ongoing synaptic shifting and/or dendritic growth in adult fish (Johns, 1981). Consistently, this dendritic label was no longer confined to two discrete bands after nerve injury, but rather dispersed throughout the entire inner plexiform layer. The implication of this shift as well as a potential re-establishment of the naïve pattern upon functional recovery is currently unknown.

The time course of GFP induction in the injured optic nerve differed slightly from its retinal expression. At 2 dpi, prominent GFP fluorescence was already visible at the optic nerve lesion site while hardly any GFP was yet detected within the retina. This pattern is consistent with foremost transport of GAP-43 to the injured axonal tip to ensure induction of a regenerative response (Skene and Willard, 1981; Skene et al., 1986). Accordingly, even single pioneering axons regenerating into the distal optic nerve were easily identified in cleared optic nerve wholemounts at 2 dpi, indicating rapid induction of regenerative growth in zebrafish. As quite a few axons had already grown past the optic chiasm by 4 dpi (this study) and reportedly reach the optic tectum by 7–8 dpi (Kaneda et al., 2008; Wyatt et al., 2010; Zou et al., 2013), regeneration is faster than in goldfish (Matsukawa et al., 2004). Visual function in zebrafish is at least partially recovered at 14 dpi (Kaneda et al., 2008). GAP-43 expression is reportedly down-regulated upon target contact and functional recovery (Bormann et al., 1998), correlating with our observed decline in GAP43 mRNA from 8–16 dpi. Comparably, we detected reduced GFP intensity proximal to the lesion site at 14 dpi, which is congruent with down-regulation of GFP expression. The fugu GAP43 promoter of the transgenic construct is expected to be turned off similarly to the internal one. GFP protein would then gradually diminish in regenerated axons due to ongoing degradation, which could be a possible explanation for the observed punctuate staining proximal to the lesion site. The axons are nevertheless healthy as we could detect restoration of visual function (data not shown). In addition, the distal parts of regenerating axons were detectable beyond 14 dpi, which enables easy tracing and analysis of individual axons throughout the entire regeneration process. Therefore, GAP43:GFP zebrafish (or other suitable transgenics, in combination with our established methods of tissue clearing and *in vitro* culturing, will facilitate the use of adult zebrafish in RGC axon regeneration research. Dissociated cell cultures could replace the predominantly used retinal explants, which are more laborious, less yielding and their axonal growth is less accurate to quantify. In addition, the easy identification of GAP43:GFP RGCs in culture will allow the fast *in vitro* screening of compound or expression libraries for growth promoting molecules as well as detailed molecular analysis of regeneration-associated processes such as cytoskeletal rearrangements or growth cone formation. Moreover, our *in situ* visualization of axonal regeneration within wholemount optic nerves can now be combined with genetic knockdown or pharmacological inhibitors to quickly analyze the *in vivo* relevance of specific proteins and signaling cascades for axonal growth and pathfinding after injury. Similarly, molecular processes required for proper myelination of regenerated axons could be investigated using transgenic zebrafish with reporter expression in oligodendrocytes (Jung et al., 2010; Münzel et al., 2012), which cannot yet be addressed in mammals due to insufficient regeneration. Overall, the presented methodologies provide exciting new possibilities to investigate the molecular mechanisms underlying successful CNS regeneration in zebrafish.

## Conflict of Interest Statement

The authors declare that the research was conducted in the absence of any commercial or financial relationships that could be construed as a potential conflict of interest.
